# Role of the Single-Stranded DNA–Binding Protein SsbB in Pneumococcal Transformation: Maintenance of a Reservoir for Genetic Plasticity

**DOI:** 10.1371/journal.pgen.1002156

**Published:** 2011-06-30

**Authors:** Laetitia Attaiech, Audrey Olivier, Isabelle Mortier-Barrière, Anne-Lise Soulet, Chantal Granadel, Bernard Martin, Patrice Polard, Jean-Pierre Claverys

**Affiliations:** 1 Centre National de la Recherche Scientifique, LMGM-UMR5100, Toulouse, France; 2 Université de Toulouse, UPS, Laboratoire de Microbiologie et Génétique Moléculaires, Toulouse, France; Université Paris Descartes, INSERM U1001, France

## Abstract

Bacteria encode a single-stranded DNA (ssDNA) binding protein (SSB) crucial for genome maintenance. In *Bacillus subtilis* and *Streptococcus pneumoniae*, an alternative SSB, SsbB, is expressed uniquely during competence for genetic transformation, but its precise role has been disappointingly obscure. Here, we report our investigations involving comparison of a null mutant (*ssbB* ^−^) and a C-ter truncation (*ssbB*Δ*7*) of SsbB of *S. pneumoniae*, the latter constructed because SSBs' acidic tail has emerged as a key site for interactions with partner proteins. We provide evidence that SsbB directly protects internalized ssDNA. We show that SsbB is highly abundant, potentially allowing the binding of ∼1.15 Mb ssDNA (half a genome equivalent); that it participates in the processing of ssDNA into recombinants; and that, at high DNA concentration, it is of crucial importance for chromosomal transformation whilst antagonizing plasmid transformation. While the latter observation explains a long-standing observation that plasmid transformation is very inefficient in *S. pneumoniae* (compared to chromosomal transformation), the former supports our previous suggestion that SsbB creates a reservoir of ssDNA, allowing successive recombination cycles. SsbBΔ7 fulfils the reservoir function, suggesting that SsbB C-ter is not necessary for processing protein(s) to access stored ssDNA. We propose that the evolutionary raison d'être of SsbB and its abundance is maintenance of this reservoir, which contributes to the genetic plasticity of *S. pneumoniae* by increasing the likelihood of multiple transformation events in the same cell.

## Author Summary

Natural genetic transformation can compensate for the absence of sexual reproduction in bacteria, allowing genetic diversification by frequent recombination. In many species, transformability is a transient property relying on a specialized membrane-associated machinery for binding exogenous double-stranded DNA and internalization of single-stranded DNA (ssDNA) fragments extracted from exogenous DNA. Subsequent physical integration of internalized ssDNA into the recipient chromosome by homologous recombination requires dedicated cytosolic ssDNA–processing proteins. Here, we document the roles in the model transformable species *Streptococcus pneumoniae* of one of these processing proteins, SsbB, a paralogue of SsbA the ssDNA–binding protein essential for genome maintenance in bacteria, which is expressed uniquely in cells competent for genetic transformation. We show that SsbB is highly abundant, potentially allowing the binding of ∼1.15 Mb ssDNA (half a genome equivalent); that it participates in the processing of ssDNA into recombinants; that it protects and stabilizes internalized ssDNA; and that, at high DNA concentration, it is of crucial importance for chromosomal transformation whilst antagonizing plasmid transformation. We conclude that SsbB creates a reservoir of ssDNA, presumably allowing multiple transformations in the same cell, and that *S. pneumoniae* has evolved SsbB to optimize chromosomal transformation, thereby contributing to its remarkable genetic plasticity.

## Introduction

Natural genetic transformation can compensate for the absence of sexual reproduction in bacteria and allows genetic diversification by frequent recombination. Two recent studies illustrated the remarkable ability of the transformable species *Streptococcus pneumoniae*, a Gram positive human nasopharyngeal commensal and respiratory pathogen, to evolve rapidly [1], [2]. The former study revealed that the genome of a strain that colonized a single child received an estimated 23 recombinational replacements over 7 months, resulting in the substitution of 7.8% of the genome [1]. In the latter study, 615 recombination events varying in size from 3 bp to 72,038 bp, with a mean of 6.3 kb, were detected through sequencing of 240 pneumococcal isolates of the same lineage [2]. These events occurred over about 40 years.

Pneumococcal transformation requires the development of competence, which relies on the transient expression of a specific set of genes encoding proteins necessary for binding of exogenous double-stranded DNA (dsDNA), for internalization of single-stranded DNA (ssDNA) fragments extracted from donor dsDNA [3], and for homologous integration of ssDNA into the chromosome [4], [5]. Expression of these genes is induced in response to an unmodified extracellular heptadecapeptide [6], called CSP (for Competence Stimulating Peptide), and is under the tight control of a complex regulatory circuit [7], [8].

Despite maintenance of intact genetic information after internalization into competent cells, ssDNA fragments reextracted from transformed cells soon after uptake have much less transforming activity than the parental dsDNA and are therefore described as being ‘in eclipse’ [9]. This transient loss of activity is accounted for by a reduced uptake of ssDNA compared to dsDNA [10]. Donor genetic information emerges from eclipse as integration of transforming ssDNA into recipient dsDNA proceeds. While in eclipse, ssDNA is recoverable from transformed cell lysates as a nuclease-resistant nucleoprotein complex [11]. ssDNA in eclipse complex (EC) is distinguishable from nucleotides and from naked ss or dsDNA by hydroxylapatite (HAP) chromatography [12]. EC was reported to contain a single competence-induced protein with an apparent molecular mass of 15.7–19.5 kDa [13], [14], identified using Western blotting as SsbB [15]. This 131 residue protein is, with SsbA (156 residues), one of two paralogous ssDNA-binding proteins present in *S. pneumoniae* [16]. Whilst the essential *ssbA* gene [17] is constitutively expressed, *ssbB* is specifically induced during competence [4], [5]. *Bacillus subtilis* also possesses two SSBs, but both are induced in competent cells [16]. Apart from its induction at competence and its cofractionation with transforming ssDNA in *S. pneumoniae*, little is known regarding the role(s) of SsbBs in transformation. As EC ssDNA was shown to be resistant to nucleases [11], it was tempting to attribute a protective role to SsbB [15]. However, reduction in chromosomal transformation due to *ssbB* inactivation appeared variable in both species. In *S. pneumoniae* a 10- to 30-fold reduction was initially reported [18], but subsequent studies only found a 3- to 5-fold reduction [15], [19], [20]. Similarly, in *B. subtilis* the transformation defect resulting from *ssbB* (previously called *ywpH*) inactivation varied from 5–10 [21], [22] to up to 50-fold [23].

To characterize the role of SsbB in DNA transformation, we investigated the impact of *ssbB* mutations on chromosomal and plasmid transformation. We show that SsbB plays a direct role in the stabilization of internalized ssDNA and that its cellular concentration is adjusted so as to handle very large quantities of ssDNA. ssDNA stabilization is of particular importance when high concentrations of exogenous DNA are available, as revealed by the diminution in the absolute number of transformants at the highest concentration used. In contrast, our results indicate quite surprisingly that the absence of SsbB facilitates plasmid transformation at high DNA concentration. In view of these findings, we propose that the evolutionary raison d'être of pneumococcal SsbB is to maintain a reservoir of internalized ssDNA to permit successive rounds of chromosomal transformation allowing the formation of new combinations of mutations, thereby directly contributing to the genetic plasticity of this species.

## Results

### SsbB Cellular Content and Importance for Chromosomal Transformation

**Figure 1:**
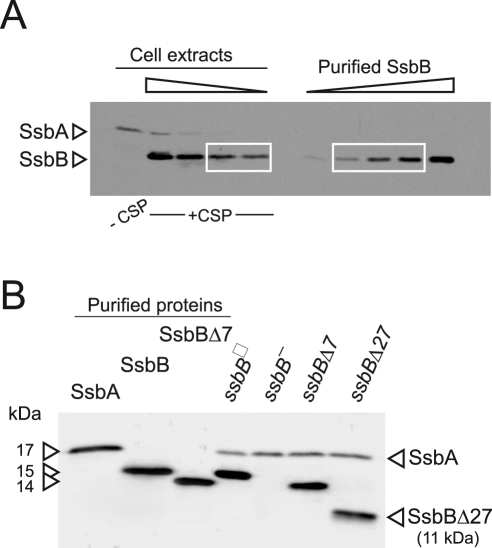
Western blot analysis of *S. pneumoniae* SsbA and SsbB proteins. (A) Quantification of SsbB in competent cells. For preparation of cell extracts, 20 ml culture in C+Y medium at OD_550_ 0.13 (∼2.1 10^8^ cfu ml^−1^) were divided into two equal parts, one of which received CSP (25 ng ml^−1^), and both were incubated for 12 min at 37°C. Total cells were then collected by centrifugation. Western blotting was as described in Materials and Methods. Volumes of total cell extracts, from left to right: 20 µl (−CSP) and 10, 5, 2.5 and 1.25 µl (+CSP). Amounts of purified SsbB, from left to right: 3.1, 6.3, 12.5, 25 and 50 ng. White rectangles identify cell extract samples and the corresponding purified protein standards used for SsbB quantification. SsbB amounts calculated for 2.5 and 1.25 µl extracts were 30.0 and 14.9 ng, respectively, resulting in an estimate of 70,799±300 SsbB molecules per cell, considering a cell density in the culture of ∼2.7 10^8^ cells ml^−1^ (because ∼30% of daugther cells remain attached to each other after completion of cell division; our unpublished observations). (B) Western-blot analysis of competent *ssbB* ^+^, *ssbB* ^−^, *ssbB*Δ*7* and *ssbB*Δ*27* cells. 5.3 µl of total extracts (corresponding to 235 µl culture at OD_550_ 0.15) were analyzed as described above. Strains used: R1501 (*ssbB* ^+^), R2294 (*ssbB* ^−^), R2081 (*ssbB*Δ*7*) and R2082 (*ssbB*Δ*27*). Purified *S. pneumoniae* SsbA (50 ng), SsbB (200 ng) and SsbBΔ7 (200 ng) proteins were used as standard.

We first wished to establish the cellular amount of both SSBs in competent pneumococcal cells. Because SsbA and SsbB share a similar, though not identical, mode of binding [24], this information is relevant to indicate whether these proteins are likely to play a significant role in the processing of internalized ssDNA. Western-blotting with crude extracts of competent cells was used to quantify SsbB and SsbA through comparison with known quantities of purified SsbA and SsbB (Figure 1A and Figure S1). The estimated number of SsbA and SsbB molecules per cell is ∼3,500 and ∼70,000, respectively. While barely detectable in non-competent cells, SsbB thus appears to be ∼20-fold more abundant than SsbA during competence. Assuming binding in the 65 mode [24], SsbB could potentially cover ∼1.15 Mb ssDNA. It is of note that the abundance of SsbB is fully consistent with a previous estimate that the competence-induced EC-bound protein represented as much as 6.4% of total proteins synthesized in competent cells [14].

To assess the importance of SsbB for chromosomal transformation, the *ssbB* gene was inactivated by *mariner* transposon insertion mutagenesis (generating mutation *ssbB*::*spc2* ^C^ hereafter called *ssbB* ^−^; Figure 1B) and mutants of SsbB harbouring a 7 or 27-residue C-ter deletion were constructed as described in Materials and Methods. SsbB C-ter truncations were constructed because of the documented importance of the acidic tail, DDD(I/L)PF, of *Escherichia coli* and *B. subtilis* SSBs for specific interactions with protein partners involved in DNA metabolism [25], [26] and because SsbB also harbours an acidic tail, EEEELPF [16]. Western-blotting confirmed the truncation of SsbB in the two mutants (Figure 1B). We then compared chromosomal transformation frequencies, scoring transformants resistant to streptomycin (Sm^R^; *rpsL41* point mutation) with R304 donor DNA (0.5–2 µg ml^−1^), in wild type, *ssbB* ^−^, *ssbB*Δ*7* and *ssbB*Δ*27* cells (Table 1). In a series of experiments with two different strains carrying the same minitransposon insertion, inactivation of *ssbB* reduced transformation 4.8 to 13.5 fold. The reason for this unusual variability will become clear in the last section of Results. In parallel, a 2.2 to 3.6 fold reduction in Sm^R^ frequency was observed in *ssbB*Δ*7* cells compared to wildtype cells (Table 1). Compared to *ssbB^−^* cells, transformation frequencies appeared significantly higher in *ssbB*Δ*7* cells suggesting that SsbBΔ7 exhibits residual activity. On the other hand, Sm^R^ frequency appeared similar in *ssbB*Δ2*7* and *ssbB^−^* cells, suggesting that SsbBΔ27 is inactive (Table 1, experiment IV). The latter mutant protein was not further characterized.

**Table 1:** Fold reduction in chromosomal transformation frequency^1^ in *ssbB* ^−^, *ssbB*Δ*7*, and *ssbB*Δ*27* cells^2^.

Experiment^3^	*ssbB* ^−^	*ssbB*Δ*7*	*ssbB*Δ*27*
**I**	13.5±5.4^4^	3.4±1.4	-5
**II**	4.8±1.9	2.1±0.8	-
**III**	9.5±2.3	2.2±0.5	-
**IV**	6.0±2.3	3.6±1.6	5.7±0.6
**V**	6.9±3.1	-	-

Competent cells were transformed with R304 donor DNA and transformants resistant to streptomycin (Sm^R^; *rpsL41* point mutation) were scored.

1 Absolute transformation frequencies are shown in Table S1.

2 Fold reduction in SmR transformants relative to the wildtype parent strain indicated in footnote 3.

3 Transformations have been carried out in parallel for all strains in the same experiment: strains R1818, R2646 and R2647 in experiments I–III; strains R1501, R1988, R2081 and R2082 in experiment IV; and strains R1501 and R1988 in experiment V.

4 Ratio errors calculated as indicated in Table S1.

5 Not done.

### SsbB Protects Internalized ssDNA

**Figure 2:**
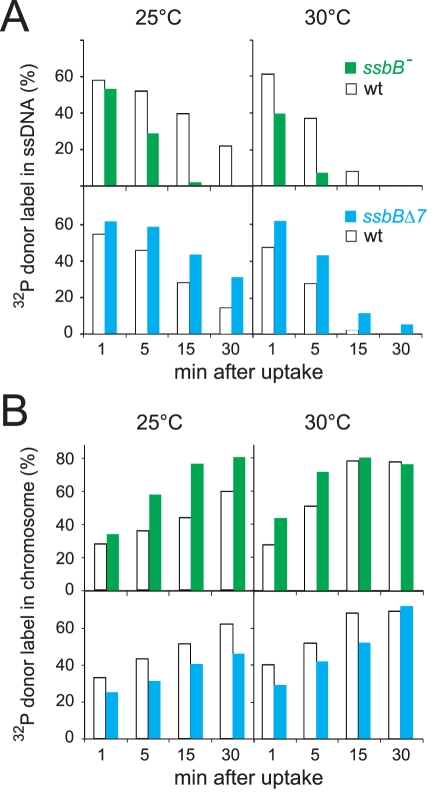
Fate of internalized ssDNA at 25°C and 30°C in wildtype and *ssbB* mutant cells. (A) Kinetics of decay of internalized ssDNA. To investigate the impact of *ssbB* mutations on the stability of internalized ssDNA, pairwise comparison experiments involving an *ssbB* mutant (*ssbB* ^−^, R2201; or *ssbB*Δ*7*, R2204) and its wildtype parent (R1521) were conducted. Competent wildtype and *ssbB* mutant cells were transformed for 3 min with a 7771-bp *S. pneumoniae* fragment uniformly labeled with ^32^P, then incubated for 1, 5, 15 or 30 min; total DNA was extracted and the fate of transforming DNA was analyzed through agarose gel electrophoresis (Materials and Methods; Figure S2). The amount of ssDNA was calculated using densitometer tracings of electrophoregrams corresponding to extracts of cells transformed at 25°C (Figure S2, panels A and C; for an example, see blue rectangle in panel A), or in parallel at 30°C, and expressed relative to total donor DNA uptake in each extract; total uptake value for each time point was normalized to the average uptake value at 25°C (Figure S2B and S2D) or 30°C. (B) Kinetics of incorporation/integration of ^32^P donor label in the chromosome. Similarly to the measurement of ssDNA label described above, the amount of donor label incorporated/integrated into the chromosome was calculated from densitometer tracings of electrophoregrams, through integration of counts in the area corresponding to chromosomal DNA (for an example, see red rectangle in Figure S2A). Same symbols as in panel A.

The physical fate of transforming DNA was then examined in competent wildtype, *ssbB*Δ*7* and *ssbB^−^* cells following exposure for 3 min to a 7771-bp homologous DNA fragment, uniformly labeled with ^32^P (Materials and Methods). Analysis of total DNA extracted after incubation at 25°C or 30°C of transformed cells for 1 to 30 min was carried out using agarose gel electrophoresis. This allows discrimination between label still associated with internalized ssDNA fragments and chromosome-associated label resulting from both homology-dependent integration and/or reincorporation via replication of ssDNA degradation products [27], [19] (Figure S2A and S2C). To evaluate the impact of *ssbB* mutations on the stability of internalized ssDNA, we choose to conduct pairwise comparison experiments involving a mutant, *ssbB* ^−^ or *ssbB*Δ*7*, and its wildtype parent. While similar uptake was achieved in each pair of strains (Figure S2B and S2D), a significant acceleration in the decay of internalized ssDNA was observed in *ssbB^−^* cells compared to wild type at both 25°C and 30°C (Figure 2A, upper part). In contrast, ssDNA appeared stabilized in *ssbB*Δ*7* cells at both temperatures (Figure 2A, lower part). As expected, ssDNA decay was accelerated in all cases at 30°C compared to 25°C. The overview of pairwise experiments conducted to compare internalized ssDNA decay in *ssbB* mutants and in the wild type established the existence of systematic and opposite biases for the *ssbB* ^−^ and *ssbB*Δ*7* mutants (Table S2). The amount of ssDNA recovered was lower in *ssbB* ^−^ than in wild type in 100% of 21 measured ratios (including the 7 values from Figure 2); and it was higher in *ssbB*Δ*7* than in wild type in 100% of 22 measured ratios (including the 8 values from Figure 2). Together with the previous demonstration that SsbB is bound to ssDNA in transformed cell extracts [15], these findings strongly suggested that SsbB protects internalized ssDNA and that the truncated SsbBΔ7 protein is still able to fulfill this role. While the latter observation was not unexpected in view of the location of the predicted ssDNA-binding site of SsbB in the N-terminal moiety [16], it indicated that the C-ter tail is not required for SsbB to readily access incoming ssDNA.

Agarose gel electrophoresis of transformed cell extracts also allowed the measurement in the same experiment of the incorporation/integration of internalized ^32^P donor label in the chromosome (Figure S2A and S2C). Our pairwise comparison approach revealed a differential behaviour of the *ssbB* ^−^ and *ssbB*Δ*7* mutants. Compared to wild type, the two mutants exhibited respectively a faster and a slower rate of incorporation/integration of donor label in the chromosome (Figure 2B). These findings were fully consistent with the observation of an accelerated rate of ssDNA degradation in *ssbB* ^−^ cells, on one hand, and of an increased protection of internalized ssDNA in *ssbB*Δ*7* cells, on the other hand.

### Impact of *ssbB* Mutations on EC

**Figure 3:**
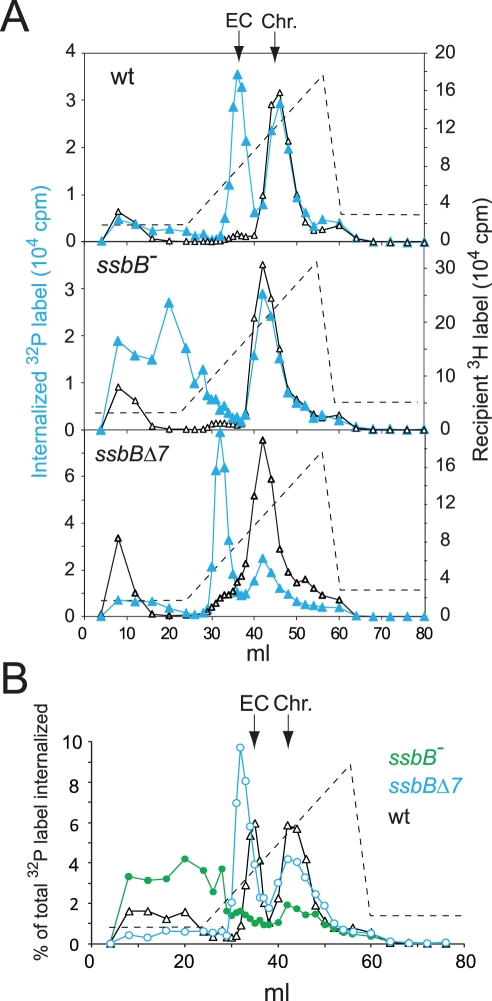
Analysis of EC in wildtype, *ssbB^−^*, and *ssbB*Δ*7* cells. (A) HAP chromatography of extracts from transformed wildtype (R2512), *ssbB*Δ*7* (R2583) and *ssbB^−^* (R2582) cells were run in parallel. Competent cells, pre-labeled with ^3^H-thymidine, were exposed for 5 min at 30°C to a mixture of unlabeled chromosomal DNA and a ^32^P-labeled 10,380-bp homologous fragment, then treated with DNase I for 1 min before lysis; preparation of cell extracts, chromatography on HAP and elution with a PB gradient were as described in Material and Methods. Blue symbols and lines, ^32^P donor label; black symbols and lines, ^3^H recipient label. Vertical arrows indicate the position of wildtype EC and chromosomal DNA (Chr.). Dotted line, PB gradient. (B) Duplicate (independent from panel A) HAP chromatography analyses of extracts from transformed wildtype (R2512), *ssbB*Δ*7* (R2583) and *ssbB^−^* (R2582) cells run in parallel. Recipient ^3^H chromosome label profiles have been omitted for clarity.

Extracts from transformed wild type and *ssbB^−^* cells were then compared using HAP chromatography to document possible changes in EC resulting from *ssbB* inactivation. Approximately half of the internalized ^32^P label was recovered as dsDNA, i.e. was eluted with 0.25–0.28 M sodium phosphate buffer (PB) [12], in both wildtype and *ssbB* ^−^ cell extracts (Figure 3). This material, which co-eluted with ^3^H-labeled recipient chromosomal DNA (Figure 3A), is known to originate from both homology-dependent integration of internalized ssDNA fragments and reincorporation of ssDNA degradation products through replication [27], [19]. The remaining part of internalized label, while eluting at the position of EC in wildtype extracts (i.e. at 0.10–0.13 M PB [12]), behaved completely differently in extracts from *ssbB^−^* cells. No label appeared at the position of nude ssDNA (i.e. at 0.17–0.18 M PB [12]) and most eluted with PB concentrations well below 0.1 M (Figure 3). As short DNA chains are known to elute at 0.07 M PB [12], this result suggested that internalized ssDNA extracted from *ssbB* ^−^ cells was in the form of short fragments. This would be consistent with its behaviour during gel exclusion chromatography on Sephacryl S400HR, which discriminates fragments by size (data not shown). This result confirmed the conclusion that *ssbB* inactivation significantly decreased the stability of internalized ssDNA, which could be the primary reason for the reduction in transformation frequency in *ssbB^−^* cells.

In support of the idea that SsbB plays a direct role in protection of ssDNA from endogenous nuclease(s), HAP chromatography analysis of extracts of transformed *ssbB*Δ*7* cells provided evidence for a direct interaction of SsbB with internalized ssDNA. Two types of changes were detected, a ∼2-fold increase in the amount of ^32^P label recovered as EC-like material combined with a shift in position of EC which eluted at a lower PB concentration than in wildtype extracts (Figure 3). The increased amount of ^32^P label in EC, and therefore of ssDNA, confirmed that internalized ssDNA is stabilized in *ssbB*Δ*7* cells. Assuming that SsbBΔ7 exhibits a mode of binding similar to that of full-size SsbB, an interpretation of this result is that more SsbBΔ7 was bound to ssDNA. Increased binding could result from a modification of the off-rate constant, k(off), of SsbBΔ7. Alternatively, deletion of the acidic C-ter might prevent interactions with processing protein(s) that are normally required to displace SsbB from ssDNA, thus slowing down ssDNA processing. As concerns the shift in EC position, we tentatively attributed it to a drastic change in the isoelectric point of the truncated protein (9.39 and 5.90 for SsbBΔ7 and SsbB, respectively) resulting from the removal of acidic residues. Whatever the explanation, we take the shift in EC position as an additional strong argument in favor of a direct interaction of SsbB with internalized ssDNA.

### Impact of *ssbB* ^−^ and *ssbB*Δ*7* Mutations on Single-Molecule Transformation

**Figure 4:**
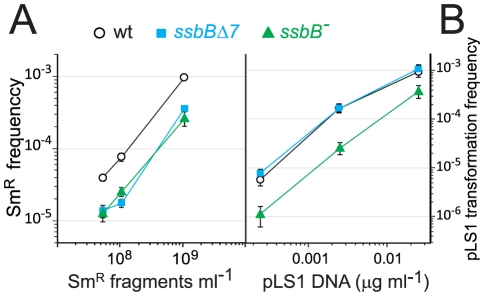
Differential effect of *ssbB* ^−^ and *ssbB*Δ*7* mutations on chromosomal and plasmid chromosomal transformation. (A) Competent cells (∼1.5 10^8^ cfu ml^−1^) of strain R1818 (*ssbB* ^+^), R2647 (*ssbB*Δ*7*) and R2646 (*ssbB* ^−^) were exposed to subsaturating concentrations (0.05 to 1 ng ml^−1^) of an 888-bp *rpsL41* fragment. Sm^R^ transformants were scored. (B) Transformation with subsaturating concentrations of pLS1 plasmid DNA (0.00025 to 0.025 µg ml^−1^) using the same competent cells as in panel A. Tc^R^ transformants were scored.

We then sought to establish whether SsbB would be required for transformation with a unique short fragment, i.e. when SsbB is in very large excess over the number of nucleotides to be protected and processed. We transformed *ssbB* ^−^, *ssbB*Δ*7* and wildtype cells with a short (888-bp or 903-bp) Sm^R^ fragment (Materials and Methods), using DNA concentrations low enough to ensure the uptake of a unique fragment per cell. Transformation with the 888-bp fragment was reduced 3.3±0.6 fold in both *ssbB^−^* and *ssbB*Δ*7* cells compared to wild type (Figure 4A). A similar reduction was observed with both mutant strains with the 903-bp fragment (3.0±0.7 fold reduction). Because the *ssbB* ^−^ and *ssbB*Δ*7* mutations had opposite effects on ssDNA stability (Figure 2 and Figure 3), we concluded that during processing of a single short molecule, SsbB is not required for protection of the internalized fragment but participates directly to the formation of homologous recombinants (transformants). We also concluded that the C-ter acidic tail of SsbB is crucial for this participation.

### SsbB and Plasmid Transformation

To further characterize the role of SsbB, plasmid transformation was investigated. While in the same experiment the *ssbB* ^−^ and *ssbB*Δ*7* mutations had a similar impact on transformation of a unique short chromosomal fragment (Figure 4A), they had a clearly different effect on plasmid pLS1 transformation (Figure 4B). Dose-response curves showed that *ssbB*Δ*7* cells behaved similarly to wild type, whilst a 6.0±0.7 fold reduction in plasmid transformation was observed with *ssbB^−^* cells at the lowest plasmid concentration. We suggest that this reduction is a direct consequence of the destabilization of internalized ssDNA observed in *ssbB^−^* cells (Figure 2), whereas SsbBΔ7 efficiently protects internalized DNA (Figure 2), thus allowing plasmid installation at normal (i.e. wildtype) frequency. These data suggest that SsbB plays no active role in plasmid installation, apart from its protective action on internalized ssDNA.

**Figure 5:**
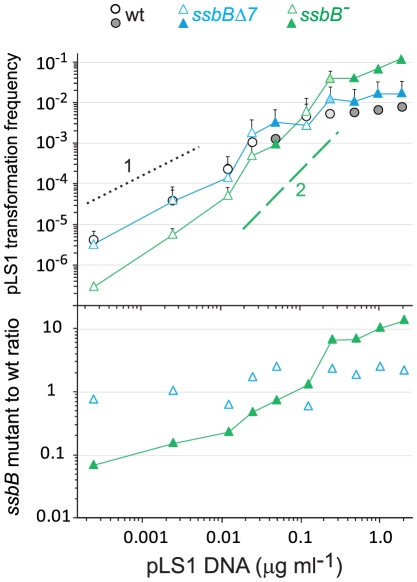
SsbB antagonizes plasmid transformation at high DNA concentration. Top panel: competent cells (∼1.1 10^8^ cfu ml^−1^) of strain R1818 (*ssbB* ^+^), R2647 (*ssbB*Δ*7*) and R2646 (*ssbB* ^−^) were exposed to pLS1 plasmid DNA. Tc^R^ transformants were scored. Data are from five independent experiments with two different pLS1 DNA preparations (I and II, distinguished by open and color-filled symbols, respectively); transformation frequencies with 0.25 µg ml^−1^ DNA correspond to average of data obtained with the two preparations (light-coloured symbols). For clarity, only half error bars are figured. Dotted and dashed lines indicate, respectively, slope of 1 and 2 (i.e. linear and quadratic dependence on DNA concentration). Bottom panel: ratio of plasmid transformants between *ssbB* mutant and wildtype cells calculated from data in top panel.

To confirm these conclusions, dose-response curves for plasmid transformation were established in parallel for wildtype, *ssbB^−^* and *ssbB*Δ*7* cells over a wider range of DNA concentrations. Whatever the concentration of plasmid DNA, *ssbB*Δ*7* and wildtype cells behaved similarly; a possible trend toward a ∼2-fold higher transformation rate in *ssbB*Δ*7* than in wildtype cells at high plasmid DNA concentrations was tentatively attributed to the increased protection of ssDNA in the former cells (Figure 5). Surprisingly, plasmid transformation frequency in *ssbB^−^* cells appeared to vary over a 100-fold range, from 10-fold lower than wild-type frequency at the lowest DNA concentrations, to 10 fold higher, at the highest concentration (Figure 5, lower panel). In the latter case, the number of plasmid transformants approached the number of chromosomal transformants for a point mutation. Such an absolute frequency was unprecedented in *S. pneumoniae*, a species in which dimeric covalently closed molecules, the most active donors for plasmid transformation, have been shown to be very inefficient [28]. We conclude from this observation that, in wildtype pneumococci, SsbB antagonizes the reconstitution of a plasmid replicon. A molecular explanation for this antagonistic role of SsbB is proposed in the Discussion.

### SsbB Boosts Chromosomal Transformation at High DNA Concentration

**Figure 6:**
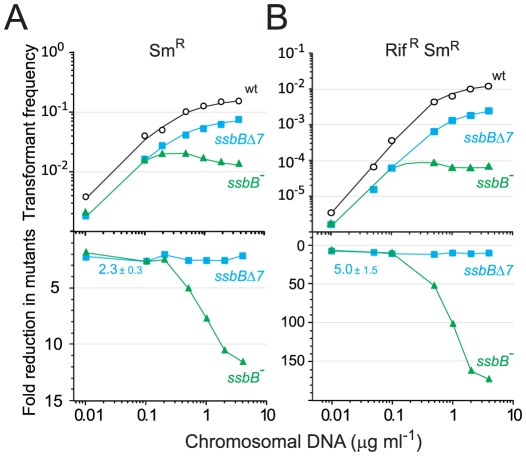
Single and double transformation frequency in wildtype, *ssbB^−^*, and *ssbB*Δ*7* cells. (A) Competent cells (∼3.0 10^8^ cfu ml^−1^) of strain R1818 (*ssbB* ^+^), R2647 (*ssbB*Δ*7*) and R2646 (*ssbB* ^−^) were exposed to various concentrations of R304 chromosomal DNA and Sm^R^ transformants were scored. Data represent the compilation of six independent experiments shown in Figure S3 (left panel). Bottom panel: ratio between the number of Sm^R^ transformants in *ssbB* mutant and wildtype cells calculated from data in top panel. (B) Same conditions as in panel A but scoring for Rif^R^ Sm^R^ double transformants. Data represent the compilation of four independent experiments shown in Figure S3 (rigth panel). Bottom panel: ratio between the number of Rif^R^ Sm^R^ transformants in *ssbB* mutant and wildtype cells calculated from data in top panel.

The finding that in competent pneumococcal cells SsbB can protect up to ∼1.15 Mb ssDNA led us to hypothesize that this protein may be very important in allowing cells to cope with high concentrations of exogenous DNA. This hypothesis was tested by investigating dose-response curves for chromosomal transformation. This analysis revealed a clear difference between *ssbB* ^−^ and *ssbB*Δ*7* cells (Figure 6). At donor DNA concentrations in the range 1–4 µg ml^−1^, Sm^R^ single and Rif^R^ Sm^R^ double transformation frequencies in *ssbB*Δ*7* cells paralleled those in wildtype cells (Figure 6, lower panel; 2.3±0.3 and 5.0±1.5 fold reduction for single and double transformants, respectively). In contrast, in *ssbB* ^−^ cells absolute Sm^R^ transformation frequencies progressively diminished with increasing DNA concentration and Sm^R^ frequency relative to wild type was reduced up to 11.6-fold at the highest DNA concentration (Figure 6, lower panel). The defect in *ssbB* ^−^ cells was confirmed through selection for Rif^R^ Sm^R^ double transformants, where a ∼170-fold reduction relative to wild type was observed (Figure 6, lower panel). It is of note that the frequencies of double Rif^R^ and Sm^R^ transformants are in all cases close to the square of the frequencies for an individual marker at the same DNA concentrations, which is what is expected for independent transformation events. The same reasoning leads to the prediction that the occurrence of a triple transformant would be 2000 to 3000-fold more frequent in the presence than in the absence of SsbB. These data thus establish that SsbB is of particular importance at high concentration of exogenous DNA and directly impacts on the likelihood of observing multiple recombination events in the same cell. Furthermore, these data provide an explanation of the unusually large fluctuations in transformation frequency of *ssbB^−^* cells compared to wild type in this (Table 1) and other studies [15], [18]–[20]. These were likely due to differences in transforming DNA concentration.

**Figure 7:**
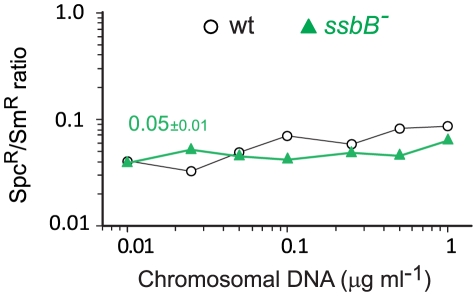
Transformation of a 8,653–nt heterology and a point mutation in wildtype and *ssbB^−^* cells. Ratio between the number of Spc^R^ (long heterology) and Sm^R^ (point mutation) transformants in strains R3055 (*ssbB* ^−^) mutant and R1818 (wild type), calculated from data in Figure S4A and S4B).

Finally, while SsbB clearly boosted chromosomal transformation at high DNA concentration, we wished to establish whether this protein also played a specific role during the processing of heterologous regions. SsbB could, for example, be required to protect from endogenous nucleases or, more generally, hide long ssDNA segments during or after heteroduplex formation. A *mariner* Spc^R^ cassette was inserted in *cps2E*, a gene in the capsule locus of strain D39; transformation of the D39 *cps2E*::*spc7* ^C^ mutation in R6 derivatives relies on integration of 8,653-nt long heterologous region including the *mariner* cassette (Materials and Methods). In wildtype cells, the heterologous region transformed with a 10-fold reduced frequency compared to the Sm^R^ point mutation (Figure 7). A similar reduction was observed in *ssbB^−^* cells; irrespective of DNA concentration, the Spc^R^/Sm^R^ ratio appeared constant (Figure 7). Therefore, SsbB plays no specific role in processing of long heterologies. On the other hand, these data confirmed a reduction not only in the relative frequency of Sm^R^ (11.3-fold; Figure S4C) or Spc^R^ (15.0-fold; Figure S4D) transformants in *ssbB* ^−^ cells compared to wild type, but also in the absolute number of Sm^R^ or Spc^R^ transformants (Figure S4A and Figure S4B) at high DNA concentration.

## Discussion

**Figure 8:**
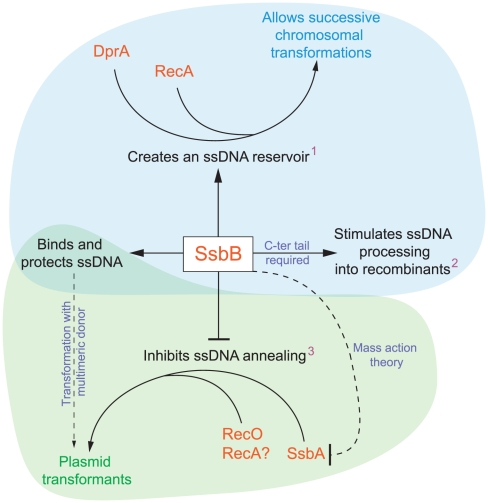
Diagrammatic representation of the roles of SsbB in competent cells of *S. pneumoniae*. SsbB binds and protects internalized ssDNA, which creates a reservoir (^1^at high donor DNA concentration) that can be accessed by DprA and RecA [20]. This reservoir then allows successive chromosomal transformations in the same cell, which directly impacts on the generation of genetic diversity. SsbB also plays a direct, yet undetermined role in the processing of ssDNA into recombinants (^2^independently of the amount and length of ssDNA internalized), for which the C-ter tail of the protein is required. Finally, SsbB antagonizes plasmid transformation (^3^at high concentration of donor DNA). We suggest that while SsbA may interact with RecO and favor reconstitution of an intact plasmid replicon through RecO (or RecA) dependent single-strand annealing, SsbB does not because its Cter differs from that of SsbA; as SsbB is ∼20-fold more abundant than SsbA, mass action is likely to result in SsbB preferential binding, thus preventing annealing and therefore plasmid reconstitution. Chromosomal and plasmid transformation are distinguished respectively by blue and green zones.

Our investigation uncovers several role(s) for SsbB in pneumococcal transformation. First, we show that inactivation of *ssbB* reduces the stability of internalized ssDNA, while SsbBΔ7 stabilizes ssDNA (Figure 2 and Figure 3). Second, we demonstrate that independently of its protective role, SsbB contributes to ssDNA processing, a function SsbBΔ7 cannot fulfill (Figure 4A). Third, we find that the absence of SsbB, while lowering plasmid transformation at low DNA concentration (Figure 4B), strongly stimulates it at high concentration of plasmid DNA (Figure 5). Fourth, we obtain no evidence that SsbB plays any specific role in processing of a 8,653-nt heterologous region (Figure 7), but SsbB is particularly important at high concentration of chromosomal DNA, possibly accounting for its abundance (∼70,000 molecules per cell; Figure 1A and Figure S1); absolute numbers of chromosomal transformants progressively diminish in *ssbB* ^−^ cells with increasing DNA concentration (Figure 6 and Figure S4) and the presence of SsbB increases transformation of two independent point mutations by up to 170 fold in wild type cells (Figure 6). Taken together, our data suggest that *S. pneumoniae* evolved the competence-induced ssDNA-binding protein SsbB to play several specific roles optimizing chromosomal transformation (Figure 8).

### SsbB Participates to the Processing of ssDNA into Recombinants

As *ssbB* ^−^ and *ssbB*Δ*7* cells exhibit a similar ∼3.3-fold deficit in transformation compared to wild-type cells when a unique 888-bp fragment is internalized (Figure 4A), whilst the *ssbB* ^−^ and *ssbB*Δ*7* mutations had opposite effects on ssDNA stability (Figure 2 and Figure 3), we first concluded that SsbB was not required for protection of the short internalized fragment but stimulates ssDNA processing into recombinants (Figure 8). It is of note that the absence of protective role of SsbB under these conditions could be accounted for by the direct loading of DprA and RecA on ‘nascent’ ssDNA, i.e. on ssDNA just emerging from the transmembrane channel [16]. We further concluded that the C-ter acidic tail of SsbB is crucial for its participation to ssDNA processing. In view of the documented key role of the acidic C-ter of *B. subtilis* SsbA [26] and *E. coli* SSB [25], it is tempting to speculate that SsbB C-ter is involved in specific interactions with other processing protein(s).

It is known that short dsDNA is cut upon binding at the cell surface [29], halving the average size of internalized ssDNA. The observed deficit thus indicates that SsbA cannot substitute for SsbB in the processing of a ∼450 nucleotide fragment, despite the fact that occluding the entire fragment would require only ∼28 or 50 SSB molecules, respectively, in the 65 or 35 mode of binding [24].

Intriguingly, a ∼2.3-fold deficit is also observed at low concentration of chromosomal DNA (Figure 6). Because nonspecific cutting at the cell surface occurs on large DNA at mean spacings that are greater than 7 kb [29], fragments taken up from chromosomal DNA are presumably >15-fold larger than with an 888-bp donor. This suggests the deficit is essentially independent of the length of ssDNA to be processed, and therefore probably of the number of SsbB molecules bound. Thus, the type of event SsbB could be involved in during processing may occur only once per fragment. SsbB could for example be required to target a processing protein to the displaced recipient strand, to trigger its specific cleavage and/or degradation.

### Protection of Internalized ssDNA by SsbB

Inactivation of *ssbB* negatively impacts the stability of internalized ssDNA, as revealed by analyzing the fate of ^32^P-labeled donor DNA using agarose gel electrophoresis (Figure 2). HAP chromatography of extracts of transformed *ssbB^−^* cells provided an independent confirmation of this destabilization, as essentially no donor label was eluted at the position of EC, the nucleoprotein complex normally found in wildtype cells (Figure 3). Thus, while some SsbA was detected at the position of EC [15], which raised the question as to whether SsbA is also involved in processing of incoming ssDNA [16], the present finding suggests that no other protein, including SsbA, can substitute for SsbB. The acidic tail of SsbB does not appear to be required for protection of internalized ssDNA since the *ssbB*Δ*7* mutation increased the half-life of internalized ssDNA (Figure 2) and resulted in more ssDNA present within EC (Figure 3). Interestingly, SsbBΔ7 EC eluted earlier, a shift we attribute to the deletion of acidic residues resulting in a severe change in the isoelectric point of the protein. We consider this result as formal proof that SsbBΔ7 is bound to internalized ssDNA. Altogether, these findings are consistent with a protective role of SsbB against endogenous nuclease(s). We propose that the deficit of chromosomal transformants in *ssbB* ^−^ cells at high DNA concentration (Figure 6 and Figure S4), as well as of plasmid transformants at low DNA concentration (Figure 4B and Figure 5), results directly from the absence of protection of internalized ssDNA by SsbB.

While arguing in favor of a direct protective role of SsbB, the present data appear puzzling in view of a previous finding that ssDNA is almost immediately degraded when internalized in *dprA* or *recA* mutant cells [19]. The latter observation necessarily implies that in the absence of DprA (or RecA), SsbB cannot access to and protect ssDNA. Possible explanations to this paradox have been proposed [16], [20] but none has received support yet.

### Significance and Possible Consequences of SsbB Abundance

#### Maintenance of an ssDNA reservoir and fratricide

The amount of SsbB in competent *S. pneumoniae* cells measured in this study (∼70,000 molecules per cell) appears adjusted so as to handle up to ∼1.15 Mb ssDNA (in the 65 mode of binding). Altogether, these data support the previous suggestion that SsbB maintains a reservoir of internalized ssDNA, which is accessible to DprA and RecA [20], [16]. As SsbBΔ7 appears fully functional with respect to SsbB reservoir function (Figure 6), we conclude that the acidic tail of SsbB is probably not required for DprA and RecA to access the ssDNA reservoir (Figure 8). SsbB absolute abundance in competent cells could be of particular importance in the context of fratricide, which involves the lysis of non-competent cells by attacker competent pneumococci [30], [31]. Lysis may result in a sudden local surfeit of DNA. Should large amounts of released DNA be internalized, SsbB makes it possible to store most of it.

#### An ssDNA reservoir for genetic plasticity but also for chromosome repair?

The first striking consequence of SsbB absence is the 11-to-15 and up-to-170 fold reduction relative to wild type in single and double transformants at high DNA concentrations (Figure 6 and Figure S4). This observation provides direct support to the view that SsbB-dependent storage of internalized ssDNA is crucial for *S. pneumoniae* genetic plasticity by allowing successive transformations in the same cell (Figure 8). On the other hand, if SsbB abundance directly reflects the uptake potential of competent pneumococci, i.e. if each cell could internalize up to half a genome equivalent of ssDNA, then the probability of repair of chromosome damages using internalized ssDNA as template [32] would become highly significant. Thus, while the original proposal of template-directed repair [32] was called into question [33], in view of *S. pneumoniae* SsbB abundance it would be interesting to reinvestigate the maximal amount of DNA that can effectively be taken up when cells are exposed to high concentrations of donor DNA.

#### Prevention of abortive processing

The second striking consequence of SsbB absence is the paradoxical decrease in absolute transformants at high concentrations of donor DNA (Figure 6 and Figure S4). This observation suggests that a key processing protein becomes limiting in the presence of large amounts ssDNA, progressively slowing down and eventually inhibiting the formation of chromosomal recombinants. One of the roles of SsbB could thus be to prevent ‘unproductive’ binding of essential component(s) of the DNA processome by temporarily ‘masking’ excess internalized ssDNA.

#### Interplay of SsbB and SsbA

SsbB abundance relative to SsbA raises the question of the interplay of the two SSBs. As SsbB binds to ssDNA with an affinity that is similar or higher than that of SsbA [34], mass action theory would predict that SsbB will substitute for SsbA throughout the cell. This raises intriguing questions. For example, would the displacement of SsbA from chromosome replication intermediates be detrimental? Or is SsbB kept away from replication forks, and if so, how? Do SsbA and SsbB display similar or different protein partnerships? Answering these and other questions would help understand how pneumococcal cells deal with the sudden massive production of SsbB during competence.

### Plasmid Transformation in *S. pneumoniae*

#### At low concentration of plasmid donor DNA

Because the mechanism of DNA uptake converts the donor dsDNA to ssDNA fragments, establishing a plasmid by transformation in *S. pneumoniae* is complex. The mechanism used depends on the nature of the donor molecule, monomeric or multimeric [28], and on the plasmid DNA concentration. Linear dependence (first-order kinetics) was previously observed with dimeric donor molecules [28]. Such kinetics was tentatively explained as reconstitution of a replicon relying on the uptake of a unique dimeric strand, followed by initiation of plasmid replication to synthesize its complement and subsequent recircularization to regenerate an intact monomeric replicon [35]. Dose-response curves in wildtype and *ssbB*Δ*7* cells exhibited a linear-dependence on concentration at low plasmid DNA concentration (Figure 5), interpreted as reflecting uptake of a unique strand from a multimeric donor. The presence of ∼63% multimers in our pLS1 plasmid preparation (Figure S5) is consistent with this interpretation. Because it is crucial for plasmid reconstitution by the postulated mechanism that the internalized strand remains longer than a monomer throughout the process, we attribute the ∼10-fold reduction in plasmid transformation at low DNA concentration in *ssbB* ^−^ cells (Figure 4B and Figure 5) to the lack of protection of internalized plasmid DNA. We propose that under these conditions, the role of SsbB is only passive (protective). It is of note that at low DNA concentration, plasmid DNA requires SsbB-dependent protection, whilst the 8,653-nt heterology does not despite an overall longer length (Figure 7). We propose that reconstitution of a plasmid replicon by this mechanism is a slow process, hence the requirement for protection, while a faster formation of chromosomal recombinants would account for the observed non-requirement for a specific protection of long heterologies.

#### At high concentration of plasmid donor DNA

A quadratic dependence on DNA concentration (two-hit kinetics) was previously observed with monomeric donor and was taken as indicative of the annealing of ssDNA fragments that have entered the cell separately, from two monomer molecules [28]. We attribute the two-hit kinetics exhibited by *ssbB^−^* cells at high concentration of plasmid DNA (Figure 5) to this mechanism. We propose that annealing becomes particularly efficient in cells lacking SsbB and accounts for the highly efficient plasmid transformation observed with *ssbB* ^−^ cells (Figure 5), and therefore that SsbB normally antagonizes annealing in wild-type cells under these conditions (Figure 8). The observation that a C-ter truncation of SsbB had no effect on plasmid transformation whatever the concentration of DNA (Figure 4B and Figure 5) could indicate either that proteins that are required for plasmid establishment do not interact with SsbB through its C-ter or that SsbB plays no active role in the reconstitution of an intact plasmid replicon. In light of the antagonistic effect of SsbB at high plasmid concentration, we favor the latter explanation.

As concerns the molecular basis for the antagonistic effect of SsbB, this protein may prevent access of an annealing protein to internalized plasmid single strands. RecO is an obvious candidate in view of its documented role in *Escherichia coli* [36] and its involvement in plasmid transformation in *B. subtilis* [37]. We propose that in the absence of SsbB, SsbA binds internalized ssDNA and allows RecO to access and efficiently anneal plasmid strands (Figure 8). This scenario is based on the assumption that *S. pneumoniae* SsbA interacts specifically with RecO through its C-ter as its *B. subtilis* counterpart does [26] and the fact that the acidic tails of *S. pneumoniae* SsbA and SsbB differ [16].

Our observations help explain a previous remark that “…intracellular processes are very inefficient for establishing new replicons in the recipient cell, whereas they are efficient for adding DNA to the chromosome…” [28]. They resolve a long-standing paradox that naturally occurring plasmids are infrequent in *S. pneumoniae*. Thus, only 3/77 pneumococcal isolates from Europe were found to harbor one or two cryptic plasmids [38]. This scarcity was puzzling in view of the conservation of transformation in the species [39] and of the capacity of pneumococci to accept the transfer by transformation of plasmids originally isolated from other species [40]. Since our data establish that SsbB antagonizes plasmid establishment, we conclude that the competence-induced SSB paralogue, as well as pneumococcal transformation per se, have evolved to favor chromosomal transformation/homologous recombination rather than plasmid transfer (Figure 8).

### Concluding Remarks

Because SsbB stabilizes internalized ssDNA, is highly abundant during competence and, at high DNA concentration, is of crucial importance for chromosomal transformation whilst antagonizing plasmid transformation, we propose that the evolutionary raison d'être of pneumococcal SsbB and its abundance is the maintenance of a reservoir of internalized ssDNA. Thereby SsbB increases the likelihood of occurrence of multiple chromosomal transformations in the same cell by allowing subsequent processing of internalized molecules by DprA and RecA (Figure 8), after completion of a first round of recombination. SsbB could thus permit the rescue of rare beneficial mutations present in the population and/or the creation of new combinations of mutations, directly contributing to the generation of diversity and to the genetic plasticity of *S. pneumoniae*. In *B. subtilis*, SsbB abundance in competent cells [41] and the scatter in transformation defect in *ssbB* mutants [21]–[23] are reminiscent of the situation in *S. pneumoniae*. We conclude that the main role of SsbB is to optimize chromosomal transformation in *S. pneumoniae*. Whether this conclusion also applies to *B. subtilis* and more generally to other naturally transformable species remains to be established.

## Materials and Methods

### Bacterial Strains, Plasmids, and Primers

*S. pneumoniae* strains, plasmids and primers used in this study are described in Table S3. The *ssbB*Δ*7* and *ssbB*Δ*27* mutations were constructed by designing primers, respectively ssbB18 and ssbB17, designed so as to introduce a premature stop codon. PCR fragments generated with the ssbB14-ssbB18 and ssbB14-ssbB17 primer pairs were cloned into a ColE1 plasmid derivative, pR326, and the resulting plasmids (pR469 and pR470) were used as donors in transformation of strain R1501. Homology-dependent integration of plasmid pR469 and pR470 at *ssbB* created the *ssbB*Δ*7* and *ssbB*Δ*27* mutations, respectively (strains R2081 and R2082; Table S3). Western-blot characterization of the SsbBΔ7 and SsbBΔ27 mutant proteins were performed as described below; C-ter truncations of SsbB did not appear to reduce significantly the amount of mutant protein per cell (Figure 1B).

Insertions of an Spc^R^ cassette (*spc* gene) in *cps2E*, *ssbB* and *thyA* was obtained by *in vitro mariner* mutagenesis as previously described [42]. Plasmid pR412 was used as a source for the *spc* minitransposon (Table S3). Briefly, plasmid DNA (∼1 µg) was incubated with a target PCR fragment in the presence of purified *Himar1* transposase, leading to random insertion of the minitransposon within the fragment. Gaps in transposition products were repaired as described [42] and the resulting *in vitro*-generated transposon insertion library was used to transform an *S. pneumoniae* strain. Location and orientation of minitransposon insertions were determined as previously described [42] through PCR reactions using primers MP127 or MP128 in combination with either one of the primers used to generate the target PCR fragment. PCR fragments to target the *cps2E*, *ssbB* and *thyA* genes were generated using the cps2C1-cps2F1, ssb1-ssb2 and thyA1-thyA2 primer pairs (Table S3), respectively. Cassette-chromosome junctions were sequenced and the *cps2E*::*spc7* ^C^ (cassette at position +949 with respect to *cps2E* start codon), *ssbB*::*spc2* ^C^ (cassette at position +158 with respect to *ssbB* start codon) and *thyA*::*spc5* ^A^ (cassette at position +139 with respect to *thyA* start codon) insertions were retained (strains TD153, R1192 and R2512, respectively; Table S3).

Inactivation of *cps2E*, the fifth gene in the *cps* operon in strain D39, is known to abolish synthesis of the polysaccharide capsule [43]. As the D39 derivative R6 (Table S3) contains a 7,505-bp deletion of the *cps2A* to *cps2H* genes, corresponding to bp 314,740–322,244 of D39 NCTC 7466 [44], transformation of the D39 *cps2E*::*spc7* ^C^ cassette (see above) in R6 derivatives relies on integration of 8,653-nt long heterologous region including the *spc* minitransposon.

### Growth and Transformation

Stock cultures were routinely grown at 37°C in Casamino Acid Tryptone (CAT) medium to OD_550_ = 0.4; after addition of 15% glycerol, stocks were kept frozen at −70°C. CSP-induced transformation [6] was performed in C+Y medium as described previously [45], using precompetent cells treated at 37°C for 10 min with synthetic CSP-1 (100 ng ml^−1^). After addition of transforming DNA and unless otherwise indicated, cells were incubated for 20 minutes at 30°C. Transformants were selected by plating on CAT-agar supplemented with 4% horse blood, followed by selection using a 10 ml overlay containing chloramphenicol (Cm; 4.5 µg ml^−1^), erythromycin (Ery; 0.05 µg ml^−1^), kanamycin (Kan; 250 µg ml^−1^), spectinomycin (Spc; 100 µg ml^−1^), streptomycin (Sm; 200 µg ml^−1^) or tetracyclin (Tc; 1 µg ml^−1^), after phenotypic expression for 120 min at 37°C.

An 888-bp or a 903-bp fragment carrying the *rpsL41* allele, which confers resistance to Sm, used as transforming DNA were amplified from R304 chromosomal DNA using respectively the IM51-IM52 or the rpsL_7-IM52 primer pairs (Table S3).

### Labeling of Donor DNA and Analysis of the Fate of Donor DNA Label on Agarose Gel

20-ml culture (∼10^8^ cfu ml^−1^) was pre-incubated at 37°C for 3 min and treated with CSP-1 for 12 min at 37°C. Culture was then divided in two parts (9 ml each) that were further processed in parallel at 25°C or 30°C as follows. After 3 min of incubation, competent cells were exposed for 3 min to 40 µl of a 7.771-bp *S. pneumoniae* fragment, uniformly labeled with ^32^P (∼500 ng; ∼2 10^6^ cpm). This fragment was generated by PCR-amplification in the presence of [α^32^P]-dATP, using as template R800 chromosomal DNA and the BM37–AM15 primer pair (Table S3) as previously described [46] but with the Phusion polymerase (Ozyme). Uptake was terminated with DNase I (50 µg ml^−1^; 100 Kunitz Units ml^−1^) and incubation was continued. After 1, 5, 15 and 30 min incubation, 2 ml samples were taken and cooled down by addition of 8 ml cold CAT medium. Cultures were then centrifuged for 10 min at 10,000 g to harvest cells. The pellet was resuspended in 200 µl SEDS containing RNAse A (20 µg ml^−1^) and cells were lysed as described [46]. Two phenol extractions followed by ethanol precipitation were used to recover total DNA which was resuspended in 50 µl Tris buffer (10 mM, pH 8.5). DNA was subjected to overnight electrophoresis (28 V cm^−1^) on 15-cm-long 1% agarose gel in Tris-acetate/EDTA buffer. Gels were dried for 2 hr at 53°C before exposure to a Phosphorimager screen (Fuji Photo Film). Electrophoregrams were analyzed using the MultiGauge software (Fujifilm).

### HAP Chromatography Analysis of Total Extracts from Transformed Cells

A thymine-requiring strain was used as recipient to allow labeling of recipient DNA through incorporation of ^3^H-thymidine during growth of precompetent cells in C+Y medium containing 150 µg ml^−1^ cold thymidine + 75 ng ml^−1^ ^3^H-thymidine (PerkinElmer NET355; Sp. Act. 20 Ci mmol^−1^). A 400-ml culture of *thyA* mutant strains R2512, R2582 and R2583 (Table S3) at an OD_550_ = 0.136 in C+Y medium was treated with 40 µg of CSP-1 at 30°C for 15 min and exposed to 978 µl of a mixture containing 72 µl of a uniformly ^32^P-labeled 10,380-bp fragment (93 µg ml^−1^) prepared as described above but with BM37-BM112 primer pair (Table S3) and 906 µl of cold chromosomal DNA (555 µg ml^−1^); the latter was added to ensure uptake and processing of significant amounts of DNA into each cell in the transformed culture; the specific activity of the mix used for transformation of wildtype, *ssbB* ^−^ and *ssbB*Δ*7* cells in Figure 3A was respectively 88,097, 70,078 and 62,134 cpm µl^−1^. After 5 min exposure to transforming DNA, DNase I was added (5 µg ml^−1^+10 mM MgCl_2_; final concentrations) for 1 min and the culture was then rapidly cooled before centrifugation at 5,000 g for 15 min. The pellet was washed with 30 ml cold SSC (SSC is 0.15 M NaCl, 0.015 M sodium citrate) and resuspended in 4 ml lysis buffer containing 0.1 M NaCl, 0.05 M Tris-HCl (pH 7.5), 0.01 M EDTA, 0.5% Sarkosyl, 0.1% Triton X-100, 1 mM phenylmethylsulfonyl fluoride, 50 µg ml^−1^ RNase A, and 10% glycerol. The clear lysate obtained after 10 min at 37°C was passed 20 times through a 5-cm 21G hypodermic needle to reduce viscosity. 4-ml sheared lysate (corresponding to 400 ml transformed culture) were loaded on a 5-ml HAP column (∼4-ml flowthrough collected) followed by elution with PB as previously described [15],[12]. Briefly, columns were washed with 20 ml 0.04 M PB (5 fractions collected) and developed with a 32-ml linear gradient of 0.04 M to 0.4 M PB (2 2-ml, followed by 10 1-ml and 9 2-ml fractions collected), then washed with 24 ml 0.4 ml 0.065 M PB + 2 M NaCl (6 fractions collected). 1/50^th^ of each fraction was mixed with 3 ml Ultima GOLD (Perkin Elmer) to measure ^3^H counts. ^3^H channel counts were corrected for overflow from the ^32^P channel.

### Western Blot Analysis of *S. pneumoniae* SsbA and SsbB Proteins

For quantification of SsbA and SsbB, cells from 10 ml culture were collected by centrifugation; pellets from the CSP-induced and control cultures were resuspended in 300 µl 1× Tris (10 mM, pH 8.0) -EDTA (1 mM) buffer and lysed for 10 min at 37°C after addition of 8 µl DOC (0.25%)-SDS (0.5%). 100 µl loading buffer were added and the suspension was incubated for 5 min at 85°C before loading onto a 15% acrylamide-SDS gel. Western blotting were classically performed as described in reference [15], using rabbit polyclonal antibodies raised against purified *S. pneumoniae* SsbB protein (2 hr [Figure 1A] or overnight [Figure 1B] incubation at room temperature with a 1/5000^th^ dilution). The ECL (Figure 1A) or ECL Plus (Figure 1B) Western Blotting Detection System (GE Healthcare) and a BioImager were used for signal detection (2 min exposure).

## Supporting Information

Figure S1 Quantification of SsbA in competent cells of *S. pneumoniae*. Amounts of purified SsbA, from left to right: 3.1, 6.3, 12.5, 25 and 50 ng. Volumes of total cell extracts, from left to right: 20 µl (−CSP) and 1.25, 2.5, 5 and 10 µl (+CSP). See Legend of Figure 1 for details. White rectangles identify cell extract samples and the corresponding purified protein standards used for SsbA quantification. SsbA amounts calculated for 5 and 10 µl extracts were respectively 3.2 and 7.4 ng, resulting in an estimate of 3,529±351 SsbA molecules per cell.
(EPS)

Figure S2 Fate of donor DNA label at 25°C analyzed by agarose gel electrophoresis. (A) Competent wildtype (R1521) and *ssbB^−^* (R2201) cells were transformed for 3 min with a 7771-bp *S. pneumoniae* fragment uniformly labeled with ^32^P, then incubated for 1, 5, 15 or 30 min; total DNA was extracted and the fate of transforming DNA was analyzed through agarose gel electrophoresis (Materials and Methods). Positions of chromosomal dsDNA (Chr.) and of ssDNA are indicated by a red open arrowhead and a vertical blue line, respectively. (B) Quantification of total donor DNA uptake in extracts of wild type (R1521) and *ssbB* ^−^ (R2201) cells at 25°C. The total amount of donor DNA label (i.e. label throughout the entire lane) in 1–30 min extracts was calculated using densitomer tracings of the electrophoregram shown in panel A. (C) Fate of donor DNA label in competent wildtype (R1521) and *ssbB*Δ*7* (R2204) cells at 25°C. Analysis was carried out as described for panel A. (D) Quantification of total donor DNA label in extracts of wild type (R1521) and *ssbB*Δ*7* (R2204) cells at 25°C using densitomer tracings of the electrophoregram shown in panel C. Quantification of ^32^P label in ssDNA and in chromosome was calculated using the area covered by the blue and red rectangles, respectively, as illustrated in panel A.
(EPS)

Figure S3 Dose-response curves for single and double transformation in wildtype, *ssbB*Δ*7*, and *ssbB^−^* cells. Competent cells of strain R1818 (*ssbB* ^+^; triangles), R2646 (*ssbB* ^−^; circles) and R2647 (*ssbB*Δ*7*; squares) were exposed to increasing concentrations of R304 chromosomal DNA. Single (Sm^R^) or double (Rif^R^ Sm^R^) transformants were scored (Materials and Methods). Left panel: six independent transformation experiments (respectively white, black, blue, green, yellow and red symbols) are shown. Right panel: four independent transformation experiments (respectively white, black, blue and green symbols) are shown.
(EPS)

Figure S4 Transformation of a 8,653–nt heterology and a point mutation in wildtype and *ssbB^−^* cells. (A) Competent cells of strain R1818 (*ssbB* ^+^; open circles) and R3055 (*ssbB* ^−^; green triangles) were exposed to various concentrations of TD153 chromosomal DNA and Sm^R^ (*rpsL41* point mutation) transformants were scored. (B) Scoring for Spc^R^ 8,653–nt heterology (*cps2E*::*spc7* ^C^ insertion within a 7,505-bp heterologous region; see Materials and Methods) transformants. (C) Ratio of Sm^R^ transformants between *ssbB* mutant and wildtype cells calculated from data in panel A. (D) Ratio of Spc^R^ transformants between *ssbB* mutant and wildtype cells calculated from data in panel C. The average ratio value ± s.e.m. calculated for the first three lowest DNA concentrations is indicated in panels C and D. Data in panels A and B are from four independent experiments; for clarity, only half error bars are figured.
(EPS)

Figure S5 Agarose gel electrophoresis of pLS1 plasmid DNA. The following samples were deposited on gel: lane 1, *Eco*RI-digested pLS1 DNA (2 µl sample); lane 2, non digested pLS1 DNA (2 µl); lane 3, DNA ladder; lane 4 non digested pLS1 DNA (5 µl). Quantification of the relative amount of pLS1 monomer and multimers using densitomer tracings indicated that the pLS1 DNA preparation contained ∼63% multimers. The amount of pLS1 plasmid DNA in the *Eco*RI-digested band (L) was estimated to be 50 ng through comparison with the 5000-bp marker band (32.5 ng deposited). Size of DNA ladder bands in bp (10 ng of each deposited, unless otherwise indicated): 20000, 10000, 7000, 5000 (32.5 ng), 4000, 3000, 2000, 1500 (40 ng), 1000 (12.5 ng), 500 (32.5 ng), 400 (12.5 ng), 300 (12.5 ng), 200 (12.5 ng), 75 (12.5 ng).
(EPS)

Table S1 Chromosomal transformation frequency in wildtype, *ssbB* ^−^, *ssbB*Δ*7*, and *ssbB*Δ*27* cells. Cells were transformed with R304 chromosomal DNA and Sm^R^ transformants were selected as described in the Materials and Methods.
(DOC)

Table S2 Transforming ssDNA decay analyses.
(DOC)

Table S3 Strains, plasmids, and primers used in this study.
(DOC)
