# Supplementary material for: Role of the Single-Stranded DNA–Binding Protein SsbB in Pneumococcal Transformation: Maintenance of a Reservoir for Genetic Plasticity
Source: PLoS Genet. 2011 Jun 30;7(6):e1002156. doi: 10.1371/journal.pgen.1002156 (PMC3128108; doi:10.1371/journal.pgen.1002156)
Supplement: Table S1 — Chromosomal transformation frequency in wildtype, ssbB −, ssbBΔ7, and ssbBΔ27 cells. Cells were transformed with R304 chromosomal DNA and SmR transformants were selected as described in the Materials and Methods. (DOC) [file pgen.1002156.s006.doc]

| **Table S1.** Chromosomal transformation frequency in wildtype, *ssbB*^-^, *ssbB*Δ*7* and *ssbB*Δ*27* cells. | | | | | | | | | | |
| --- | --- | --- | --- | --- | --- | --- | --- | --- | --- | --- |
|  |  |  |  |  |  |  |  |  |  |  |
|  |  |  |  |  |  |  |  | | | |
| **Experiment**^a^ |  |  |  | **wild type** |  | ***ssbB*^-^** |  | ***ssbB***Δ***7*** |  | ***ssbB***Δ***27*** |
|  |  |  |  |  |  |  |  |  |  |  |
| **I** |  | Total cfu ml^-1^ |  | 1.89 10^8^ |  | 1.80 10^8^ |  | 1.26 10^8^ |  | -^f^ |
|  |  | Sm^R^ transformants ml^-1^ |  | 3.33 10^7^ |  | 2.60 10^6^ |  | 6.99 10^6^ |  |  |
|  |  | % transformants |  | 17.6 ± 4.8^b^ |  | 1.31 ± 0.37 |  | 5.2 ± 1.7 |  |  |
|  |  | reduction in mutant^c^ |  | na^d^ |  | 13.5 ± 5.4^e^ |  | 3.4 ± 1.4 |  |  |
|  |  |  |  |  |  |  |  |  |  |  |
| **II** |  | Total cfu ml^-1^ |  | 1.59 10^8^ |  | 1.77 10^8^ |  | 1.71 10^8^ |  | - |
|  |  | Sm^R^ transformants ml^-1^ |  | 2.76 10^7^ |  | 3.22 10^6^ |  | 7.05 10^6^ |  |  |
|  |  | % transformants |  | 17.3 ± 5.0 |  | 3.6 ± 1.0 |  | 8.2 ± 2.2 |  |  |
|  |  | reduction in mutant |  | na |  | 4.8 ± 1.9 |  | 2.1 ± 0.8 |  |  |
|  |  |  |  |  |  |  |  |  |  |  |
| **III** |  | Total cfu ml^-1^ |  | 2.46 10^8^ |  | 2.90 10^8^ |  | 2.91 10^8^ |  | - |
|  |  | Sm^R^ transformants ml^-1^ |  | 2.47 10^7^ |  | 3.05 10^6^ |  | 1.30 10^7^ |  |  |
|  |  | % transformants |  | 10.0 ± 1.8 |  | 1.05 ± 0.17 |  | 4.47 ± 0.67 |  |  |
|  |  | reduction in mutant |  | na |  | 9.5 ± 2.3 |  | 2.2 ± 0.5 |  |  |
|  |  |  |  |  |  |  |  |  |  |  |
| **IV** |  | Total cfu ml^-1^ |  | 2.25 10^8^ |  | 2.82 10^8^ |  | 1.47 10^8^ |  | 2,98 10^8^ |
|  |  | Sm^R^ transformants ml^-1^ |  | 1.04 10^7^ |  | 1.98 10^6^ |  | 1.87 10^6^ |  | 2,56 10^6^ |
|  |  | % transformants |  | 4.6 ± 1.4 |  | 0.78 ± 0.19 |  | 1.28 ± 0.41 |  | 0.81 ± 0.19 |
|  |  | reduction in mutant |  | na |  | 6.0 ± 2.3 |  | 3.6 ± 1.6 |  | 5.7 ± 0.6 |
|  |  |  |  |  |  |  |  |  |  |  |
| **V** |  | Total cfu ml^-1^ |  | 2.46 10^8^ |  | 1.83 10^8^ |  | - |  | - |
|  |  | Sm^R^ transformants ml^-1^ |  | 2.47 10^7^ |  | 1.10 10^6^ |  |  |  |  |
|  |  | % transformants |  | 4.9 ± 1.6 |  | 0.71 ± 0.23 |  |  |  |  |
|  |  | reduction in mutant |  | na |  | 6.9 ± 3.1 |  |  |  |  |
|  |  |  |  |  |  |  |  |  |  |  |
|  |  |  |  |  |  |  |  |  |  |  |
| ^a^Transformations with R304 chromosomal DNA have been carried out in parallel for all strains | | | | | | | | | | |
| in the same experiment: | | |  |  |  |  |  |  |  |  |
| strains R1818, R2646 and R2647 in experiments **I-III**; | | | | |  |  |  |  |  |  |
| strains R1501, R1988, R2081 and R2082 in experiment **IV**; | | | | | |  |  |  |  |  |
| and strains R1501 and R1988 in experiment **V** | | | | |  |  |  |  |  |  |
| ^b^Numeration sampling errors calculated as recommanded by Sicard (1965) | | | | | | | |  |  |  |
| ^c^Fold reduction in transformation frequency compared to wild type | | | | | | |  |  |  |  |
| ^d^Not applicable | |  |  |  |  |  |  |  |  |  |
| ^e^Ratio errors calculated from numeration sampling errors^b^ as recommanded by Sicard (1965) | | | | | | | | | | |
| ^f^Not done |  |  |  |  |  |  |  |  |  |  |

Reference

1. Sicard AM (1965) Analyse génétique de la structure fine du locus *amiA* chez *Diplococcus pneumoniae*. Ph D Dissertation, Université de Paris, France.
