# Supplementary material for: Role of the Single-Stranded DNA–Binding Protein SsbB in Pneumococcal Transformation: Maintenance of a Reservoir for Genetic Plasticity
Source: PLoS Genet. 2011 Jun 30;7(6):e1002156. doi: 10.1371/journal.pgen.1002156 (PMC3128108; doi:10.1371/journal.pgen.1002156)
Supplement: Table S2 — Transforming ssDNA decay analyses. (DOC) [file pgen.1002156.s007.doc]

| **Table S2.**  Transforming ssDNA decay analyses. | | | |  |  |  |  |
| --- | --- | --- | --- | --- | --- | --- | --- |
|  |  |  |  |  |  |  |  |
| Systematically biased *ssbB* mutant to wild type ratios provide evidence that the absence of *ssbB* destabilizes | | | | | | | |
| internalized ssDNA (i.e. ssDNA decay is accelerated), whereas the Δ7 SsbB C-ter truncation stabilizes it | | | | | | | |
| (i.e. decay is decelerated). | | |  |  |  |  |  |
|  |  |  |  |  |  |  |  |
|  |  |  |  |  |  |  |  |
| Pairwise comparison  with wild  type of: | Experiment | | | Number of  independent measurements | *ssbB* mutant to  wild type ratio value^a^ | | Rate of ssDNA decay compared to wild type |
|  | # | T° | extraction time  after uptake |  | <1 | >1 |  |
|  |  |  |  |  |  |  |  |
| *ssbB*^-^ | 1 | 30°C | 1 | 2 | 2 | 0 |  |
|  | 2 | 25°C | 1 | 2 | 2 | 0 |  |
|  | 3^b^ | 30°C | 1,5,15 | 3 | 3 | 0 |  |
|  | 4^b^ | 25°C | 1,5,15,30 | 4 | 4 | 0 |  |
|  | 5 | 30°C | 1,5 | 2 | 2 | 0 |  |
|  | 6 | 25°C | 1,5 | 2 | 2 | 0 |  |
|  | 7 | 25°C | 1,5 | 2 | 2 | 0 |  |
|  | 8 | 25°C | 1,5 | 2 | 2 | 0 |  |
|  | 9 | 25°C | 1,5 | 2 | 2 | 0 |  |
|  | Total |  |  | 21 | 21 | 0 | Accelerated |
|  |  |  |  |  |  |  |  |
|  |  |  |  |  |  |  |  |
| *ssbB*Δ*7* | 1 | 25°C | 1 | 2 | 0 | 2 |  |
|  | 2^c^ | 30°C | 1,5,15,30 | 4 | 0 | 4 |  |
|  | 3^c^ | 25°C | 1,5,15,30 | 4 | 0 | 4 |  |
|  | 4 | 30°C | 1,5 | 2 | 0 | 2 |  |
|  | 5 | 25°C | 1,5 | 2 | 0 | 2 |  |
|  | 6 | 25°C | 1,5 | 2 | 0 | 2 |  |
|  | 7 | 25°C | 1,5,15,30 | 4 | 0 | 4 |  |
|  | 8 | 25°C | 1,5 | 2 | 0 | 2 |  |
|  | Total |  |  | 22 | 0 | 22 | Decelerated |
|  |  |  |  |  |  |  |  |
|  |  |  |  |  |  |  |  |
| ^a^For each time point, the amount of ssDNA was calculated using densitometer tracings of electrophoregrams | | | | | | | |
| corresponding to extracts from transformed cells analyzed through agarose gel electrophoresis (see Materials | | | | | | | |
| and Methods; Figure S2 and Figure 2) | | | |  |  |  |  |
| ^b^Experiment shown in Figure S2A | | |  |  |  |  |  |
| ^c^Experiment shown in Figure S2B | | |  |  |  |  |  |
