# Supplementary material for: Role of the Single-Stranded DNA–Binding Protein SsbB in Pneumococcal Transformation: Maintenance of a Reservoir for Genetic Plasticity
Source: PLoS Genet. 2011 Jun 30;7(6):e1002156. doi: 10.1371/journal.pgen.1002156 (PMC3128108; doi:10.1371/journal.pgen.1002156)
Supplement: Table S3 — Strains, plasmids, and primers used in this study. (DOC) [file pgen.1002156.s008.doc]

**Table S3.** Strains, plasmids, and primers used in this study.

| Strains | Genotype/description | Source/reference |
| --- | --- | --- |
| D39 | Serotype 2 | NCTC 7466 |
| R6 | Unencapsulated derivative of D39 | Laboratory stock |
| R246 | R800 but *hexA*::Δ3-*ermAM* | [1] |
| R304 | R800 derivative, *nov1*, *rif23*, *rpsL41*; Nov^R1^, Rif^R^, Sm^R^ | [1] |
| R800 | R6 derivative | [2] |
| R1192 | R800 but *ssbB*::*spc2*^C2^; Spc^R^ | Mathieu Bergé, Chantal Granadel |
| R1501 | R800 but Δ*comC* | [3] |
| R1521 | R1501 but *comC*::pR414 (::*luc*), Δ*comC*; Ery^R^ | [4] |
| R1818 | R1501 but *hexA*:: Δ3-*ermAM* (by transformation with R246 DNA); Ery^R^ | This study |
| R2081 | R1501 but *ssbB*::pR469, *ssbB*Δ*7*; Cm^R^ | This study |
| R2082 | R1501 but *ssbB*::pR470, *ssbB*Δ*27*; Cm^R^ | This study |
| R2201 | R1521 but *ssbB*::*spc2*^C^; Ery^R^, Spc^R^ | This study |
| R2204 | R1521 but *ssbB*::pR469, *ssbB*Δ*7*; Ery^R^, Cm^R^ | This study |
| R2294 | R1192 but *ssbB*::*kan2*^C3^; Kan^R^ | Nathalie Campo |
| R2512 | R1501 but *thyA*::*spc5*^A2^; Spc^R^ | This study |
| R2582 | R2512 but *ssbB*::*kan2*^C^ (by transformation with R2294 DNA); Kan^R^, Spc^R^ | This study |
| R2583 | R2512 but *ssbB*::pR469, *ssbB*Δ*7*; Cm^R^, Spc^R^ | This study |
| R2646 | R1818 but *ssbB*::*spc2^C^*; Ery^R^, Spc^R^ | This study |
| R2647 | R1818 but *ssbB*::pR469, *ssbB*Δ*7*; Cm^R^, Ery^R^ | This study |
| R3055 | R2646 but *ssbB*::*kan2*^C^ (by transformation with R2294 DNA); Ery^R^, Kan^R^ | This study |
| TD153 | D39 but *cps2E*::*spc7*^C^, *rpsL41*; Spc^R^, Sm^R^ | This study |
| Plasmids |  |  |
| pR326 | ColE1 derivative; Ap^R^, Cm^R^ | [5] |
| pR410 | pEM*cat* derivative carrying a Kan^R^ (*kan* gene) *mariner* minitransposon; Ap^R^, Kan^R^ | [6] |
| pR412 | pEM*cat* derivative carrying a Spc^R^ (*aad9* gene, also called *spc*) *mariner* minitransposon; Ap^R^, Spc^R^ | [7] |
| pR414 | p5.00 derivative carrying a *comC* targeting fragment adjacent to *luc*; insertion–duplication in *S. pneumoniae* generates a *comC*::*luc* (*comC^+^*) fusion; Ery^R^ | [8] |
| pR469 | ColE1 (pR326) derivative carrying an *Nco*I-*Hin*dIII *ssbB* insert (generated with ssbB14-ssbB18 primer pair; Materials and Methods); insertion–duplication in *S. pneumoniae* creates the *ssbB*Δ*7* mutation; Ap^R^, Cm^R^ | This study |
| pR470 | ColE1 (pR326) derivative carrying an *Nco*I-*Hin*dIII *ssbB* insert (generated with ssbB14-ssbB17 primer pair; Materials and Methods); insertion–duplication in *S. pneumoniae* creates the *ssbB*Δ*27* mutation; Ap^R^, Cm^R^ | This study |
| pLS1 | pMV158 derivative, replicative plasmid (rolling-circle type); Tc^R^ | [9] |
| Primers | Sequence^4^; gene; position^5^ |  |
| AM15 | TCGTATCATCAACCAAAAGATTGCT; *amiA*; +7530 | (Attaiech et al. 2008) |
| BM37 | ggaattcggaTCCTAAACTCCAAAGTTTCTGCGTC; *amiA*;  -182 | [8] |
| BM112 | CCTGCccGGGCTGAACCAGTCATCCCAG; *treP* ; +1250 | [10] |
| cps2C1 | AGAACAAAACACGATAGAAATCG; *cps2E* ; -1369 | This study |
| cps2F1 | GCCATCATAATCGCAATTTG; *cps2E* ; +2588 | This study |
| IM51 | GGACGTGCTGACAAATGTT^6^; *rpsL*; -75 | [1] |
| IM52 | GCAAGACGGTCTTGCATTGTG; *rpsL*; +774 | [1] |
| MP127 | CCGGGGACTTATCAGCCAACC; *mariner* cassette universal primer (internal to terminal IRs); outward orientation | [7] |
| MP128 | TACTAGCGACGCCATCTATGTG; *mariner* cassette universal primer (adjacent to IRL); outward orientation | [7] |
| rpsL_7 | CTCAAGAGTGTACAGGGACG; *rpsL*; -89 | This study |
| ssb1 | AAGTTGCAGTCGCAAGTGACAAGAT; *ssbB* ; -1125 | Mathieu Bergé |
| ssb2 | TTCGATGGTGATGACACCGT; *ssbB*; +1359 | Mathieu Bergé, Chantal Granadel |
| ssbB14 | GGGctcgagACGTCTACACCAGAATTGCAC; *ssbB*; +54 | This study |
| ssbB17 | ACGaagctt*tta*ACTTTCCAAGAGTTGGAATCC; *ssbB*; +291 | This study |
| ssbB18 | TTCaagctt*tta*CAAGACCAAATCTGCCAAATC; *ssbB*; +351 | This study |
| thyA1 | GCAGCAGGTGAGCTTGGTCA; *thyA*; -560 | This study |
| thyA2 | CCACGGCTGTGAATATCCTC; *thyA*; +1433 | This study |

^1R^, resistance; Ap, ampicillin; Cm, chloramphenicol; Ery, erythromycin; Kan, kanamycin; Nov, novobiocin; Rif, rifampicin; Spc, spectinomycin; Sm, streptomycin; Tc, tetracyclin.

^2C^ and ^A^ indicate respectively the co-transcribed and the reverse orientation of an inserted mini-transposon antibiotic resistance gene with respect to the targeted gene.

^3^The *spc2*^C^ *mariner* cassette insertion in *ssbB* was exchanged with the *kan* cassette by transformation of strain R1192 with plasmid pR410 DNA, selecting for Kan^R^ transformants. Cassette swapping is based on the presence of DNA homology at the borders of the synthetic *spc* and *kan* minitransposons, allowing exchange of resistance cassette genes by homologous recombination during transformation [11].

^4^Lowercase letters indicate nucleotides differing from the *S*. *pneumoniae* genome sequence, which introduce convenient restriction sites (underlined) or mutations (italics). The latter served to introduce a stop codon to generate the *ssbB*Δ*7* (primer ssbB18) and *ssbB*Δ*27* (primer ssbB17) mutations, respectively.

^5^3' oligonucleotide position is given with respect to the ATG of the corresponding gene; - and + indicate upstream and downstream, respectively.

^6^The underlined nucleotides in blue colour correct a previously published erroneous sequence.

**References**

1. Mortier-Barrière I, de Saizieu A, Claverys JP, Martin B (1998) Competence-specific induction of *recA* is required for full recombination proficiency during transformation in *Streptococcus pneumoniae*. Mol Microbiol 27: 159-170.

2. Lefèvre JC, Claverys JP, Sicard AM (1979) Donor deoxyribonucleic acid length and marker effect in pneumococcal transformation. J Bacteriol 138: 80-86.

3. Dagkessamanskaia A, Moscoso M, Hénard V, Guiral S, Overweg K et al. (2004) Interconnection of competence, stress and CiaR regulons in *Streptococcus pneumoniae*: competence triggers stationary phase autolysis of *ciaR* mutant cells. Mol Microbiol 51: 1071-1086.

4. Håvarstein LS, Martin B, Johnsborg O, Granadel C, Claverys JP (2006) New insights into pneumococcal fratricide: relationship to clumping and identification of a novel immunity factor. Mol Microbiol 59: 1297-1307.

5. Claverys JP, Dintilhac A, Pestova EV, Martin B, Morrison DA (1995) Construction and evaluation of new drug-resistance cassettes for gene disruption mutagenesis in *Streptococcus pneumoniae*, using an *ami* test platform. Gene 164: 123-128.

6. Sung CK, Li H, Claverys JP, Morrison DA (2001) An *rpsL* Cassette, Janus, for Gene Replacement through Negative Selection in *Streptococcus pneumoniae*. Appl Environ Microbiol 67: 5190-5196.

7. Martin B, Prudhomme M, Alloing G, Granadel C, Claverys JP (2000) Cross-regulation of competence pheromone production and export in the early control of transformation in *Streptococcus pneumoniae*. Mol Microbiol 38: 867-878.

8. Bergé M, Moscoso M, Prudhomme M, Martin B, Claverys JP (2002) Uptake of transforming DNA in Gram-positive bacteria: a view from *Streptococcus pneumoniae*. Mol Microbiol 45: 411-421.

9. Stassi D, López P, Espinosa M, Lacks SA (1981) Cloning of chromosomal genes in *Streptococcus pneumoniae*. Proc Natl Acad Sci USA 78: 7028-7032.

10. Guiral S, Hénard V, Laaberki M-H, Granadel C, Prudhomme M et al. (2006) Construction and evaluation of a chromosomal expression platform (CEP) for ectopic, maltose-driven gene expression in *Streptococcus pneumoniae*. Microbiology (Special Issue on Pneumococcus) 152: 343-349.

11. Caymaris S, Bootsma HJ, Martin B, Hermans PWM, Prudhomme M et al. (2010) The global nutritional regulator CodY is an essential protein in the human pathogen *Streptococcus pneumoniae*. Mol Microbiol 78: 344-360.
